# The C-Terminus of Epstein-Barr Virus BRRF2 Is Required for its Proper Localization and Efficient Virus Production

**DOI:** 10.3389/fmicb.2017.00125

**Published:** 2017-01-31

**Authors:** Takahiro Watanabe, Keiya Sakaida, Masahiro Yoshida, H. M. Abdullah Al Masud, Yoshitaka Sato, Fumi Goshima, Hiroshi Kimura, Takayuki Murata

**Affiliations:** Department of Virology, Nagoya University Graduate School of MedicineNagoya, Japan

**Keywords:** acidic cluster, BRRF2, EBV, lytic replication, phosphorylation, TGN, endosome

## Abstract

Epstein-Barr virus (EBV) is a human gammaherpesvirus associated with several malignancies. We reported previously that an EBV lytic gene product BRRF2 is involved in the maturation of progeny virus. To analyze the domain(s) needed for efficient production of progeny, we prepared a series of deletion mutants and found two functional domains in the N- and C-terminal regions by complementation assays. Immunofluorescence analyses revealed that BRRF2 lacking the C-terminal region demonstrated aberrant localization in both the nucleus and cytoplasm, whereas wild-type BRRF2 was localized predominantly in the cytoplasm. We also confirmed that wild-type BRRF2 co-localized with Rab5, an endosomal marker, at least partly. Additionally, serine 511 of BRRF2 was phosphorylated during lytic infection; however, a mutant in which the serine was substituted with alanine still augmented the yield as efficiently as did wild-type BRRF2. These results showed that the C-terminal region of BRRF2 is involved in the predominant localization of BRRF2 to the cytoplasm and in the efficient production of infectious virus.

## Introduction

The Epstein-Barr virus (EBV) is a human gammaherpesvirus that is present in most of the world’s population. EBV has DNA genome of approximately 170–180 kb that encodes more than 80 genes. Mature virus particles are transmitted via saliva, and infection occurs by invasion of the oral cavity and pharynx to establish infection in epithelial cells and B cells. In general, primary infection is asymptomatic; however, its infection causes infectious mononucleosis in a small minority of adults. Besides, EBV is associated with several proliferative disorders of lymphocytes and epithelials, including Burkitt lymphoma, Hodgkin lymphoma, chronic active EBV infection (CAEBV), and T/NK lymphoma. (Young et al., 2016).

Epstein-Barr virus can establish two types of infections: latent and lytic infections (Murata and Tsurumi, 2014). In the latent state, the viral genomic DNA exists in the nucleus in a chromatinized state (episome), in which it expresses only a limited number of viral latent genes (Lieberman, 2013). In the lytic state, all lytic genes of EBV are expressed and progeny virus particles are produced (Hammerschmidt and Sugden, 2013). Switching from the latent to lytic state is called reactivation. Reactivation can be triggered by exogenous expression of a viral immediate-early (IE) gene, BZLF1 (also called Zta, Z, ZEBRA, and EB1). BZLF1, in cooperation with another IE gene BRLF1 (also known as Rta and R), can efficiently elicit the expression of early (E) genes involved in viral DNA synthesis and late (L) gene induction. Viral DNA genomes are amplified at a discrete site in the host nucleus called replication compartments (Tsurumi et al., 2005). Several E genes (BcRF1, BVLF1, BGLF3, BFRF2, BDLF4, and BDLF3.5) form a complex in the nucleus and induce transcription of L genes in a TATT sequence (non-canonical TATA sequence)-dependent manner (Watanabe et al., 2015a; Djavadian et al., 2016; Gruffat et al., 2016). L genes encode capsid, tegument, and envelope proteins. Capsid proteins are assembled in the nucleus, and newly synthesized EBV genomic DNA is packaged into capsids (Murata, 2014). After nucleocapsid formation, the virus undergoes an envelopment, de-envelopment, and re-envelopment pathway common to alpha-herpesvirus (Mettenleiter et al., 2006; Johnson and Baines, 2011). In this model, after DNA packaging in the nucleus, the nucleocapsids bud into the inner nuclear membrane to form an enveloped particle (primary envelopment). When these particles fuse with the outer nuclear membrane, the envelope is lost, and nucleocapsids are released into the cytoplasm (de-envelopment). In the cytoplasm, nucleocapsids bud into the cytoplasmic membrane structure—likely via the *trans*-Golgi network (TGN) or endosomes—along with various tegument proteins (secondary envelopment). The virions are then released into the extracellular space by fusion of the organelle with the plasma membrane.

The BRRF2 protein of EBV is conserved among the gammaherpesvirus group, which include ORF48 of Kaposi’s sarcoma-associated herpesvirus (KSHV) and murine gammaherpesvirus 68 (MHV-68). We demonstrated previously that BRRF2 is expressed during the L phase and is localized in the cytoplasm (Watanabe et al., 2015b). Whereas BRRF2 is important for the production of progeny viruses, it is not required for viral gene expression or viral DNA replication (Watanabe et al., 2015b). Similarly, the ORF48 gene of MHV-68 appears to play a crucial role in progeny production (Qi et al., 2015). Mass-spectrometry analysis showed that BRRF2 is detectable as a phosphorylated tegument protein (Johannsen et al., 2004), but its phosphorylation is not mediated by the viral protein kinase BGLF4 (Watanabe et al., 2015b).

In this study, we generated mutant BRRF2 expression vectors to examine the domain(s) of the tegument protein important for progeny virus production. Our complementation assays revealed that the N- and C-terminal domains are required for efficient virus production. Interestingly, deletion of the C-terminal domain of BRRF2, which includes several clusters of acidic amino acids, caused aberrant intracellular localization. Furthermore, we revealed that serine 511 of BRRF2 is phosphorylated in cells, although substitution of the serine for alanine did not influence the complementation efficiency.

## Materials and Methods

### Cell Culture and Reagents

HEK293 (Delecluse et al., 1998) and HeLa (Kawashima et al., 2013) cells were maintained in Dulbecco’s modified Eagle medium (DMEM, Gibco BRL) supplemented with 10% fetal bovine serum (FBS). HEK293 cells infected with EBV BRRF2 knockout virus (Watanabe et al., 2015b) were cultured with DMEM containing 150 μg/ml hygromycin B. Akata EBV negative cells (Akata(-)), B95-8, and AGS-EBV cells (Katsumura et al., 2009) were cultured in RPMI 1640 medium containing 10% FBS.

Rabbit antibodies against BZLF1 and BALF2 were used as reported previously (Murata et al., 2009; Sugimoto et al., 2013). Mouse anti-FLAG antibody was purchased from Sigma-Aldrich, and rabbit anti-Giantin antibody was from Abcam. Rabbit Anti-tubulin, -Calnexin, and -Rab5 antibodies were purchased from Cell Signaling Technology. Mouse anti-BMRF1 antibody was purchased from Novocastra. Horseradish peroxidase-linked goat antibodies to mouse or rabbit IgG were from Amersham Biosciences.

### Plasmid Construction

**FIGURE 1:**
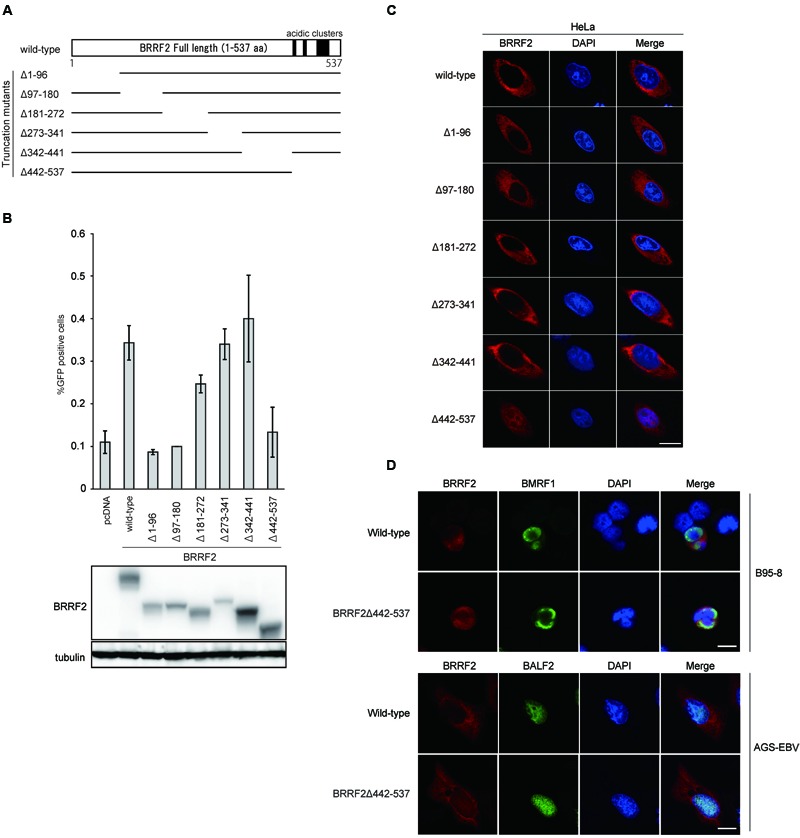
**Functional domains of BRRF2. (A)** Schematic diagram of Epstein-Barr virus (EBV) BRRF2 mutant vectors. Deletion mutants were prepared as described in Section “Materials and Methods”, using the N-terminally FLAG-tagged wild-type BRRF2 expression plasmid as a template. **(B)** Complementation assays. HEK293 cells latently infected with the BRRF2-knockout EBV BAC clone were co-transfected with BZLF1 and expression vectors harboring wild-type or deletion mutants of BRRF2. After 3 days, the supernatants were co-cultured with Akata(-) cells, and after 2 days, FACS analysis was performed to measure the viral titer by determining the GFP-positive ratio. Protein levels were examined by immunoblotting. **(C)** Intracellular localization of wild-type or deletion mutants of BRRF2. HeLa cells were transfected with vectors containing FLAG-tagged wild-type BRRF2 or the BRRF2 deletion mutants. One day after transfection, cells were fixed, permeabilized and stained with ant-FLAG (BRRF2, red) antibody and DAPI (blue) and analyzed by using confocal laser microscopy. **(D)** Intracellular localization of BRRF2 in EBV-positive cells during lytic infection. B95-8 (upper panels) or AGS-EBV (lower panels) cells were transfected with BZLF1 and BRRF2 vectors by electroporation or lipofection, respectively. After 2 days, cells were harvested and stained with anti-FLAG (BRRF2, red), BMRF1, or BALF2 (green) antibodies and DAPI (blue) and analyzed by confocal laser microscopy. The white bar indicates 10 μm.

**FIGURE 2:**
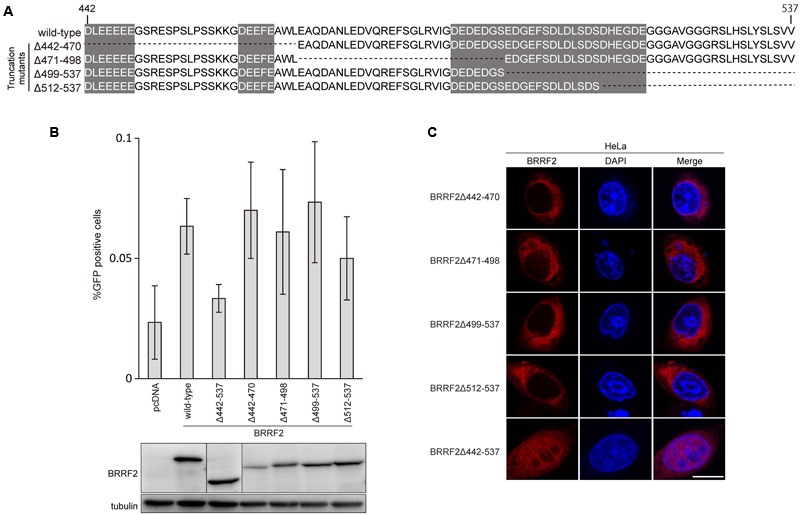
**The C-terminal region of BRRF2 regulates its cytoplasmic localization (A)** Schematic diagram of EBV BRRF2 mutant vectors. Acidic cluster motifs are indicated with shadowing. **(B)** Complementation assays. HEK293 cells carrying the BRRF2-knockout EBV BAC clone were transfected with BZLF1 and the indicated BRRF2 vectors. Virus titer was determined as in **Figure 1B**. Protein levels were examined by immunoblotting. **(C)** Intracellular localization of wild-type or deletion mutants of BRRF2. HeLa cells were transfected with vectors containing FLAG-tagged wild-type BRRF2 or the BRRF2 mutants. Cells were stained with ant-FLAG (BRRF2, red) antibody and DAPI (blue) and analyzed by using confocal laser microscopy. The white bar indicates 10 μm.

Two expression vectors, pcDNABZLF1 and pcDNAFLAGBRRF2, were reported previously (Watanabe et al., 2015b). All mutant vectors with deletions or substitutions in BRRF2 were generated by inverse PCR using Flag-tagged wild type BRRF2 expression plasmid as a template. To generate the deletion mutants shown in **Figures 1A** and **2A**, we used the following primers: for Δ1-96: 5′-TGTCTGGGTGTTATTCCAGG-3′ (forward), and 5′-CATGAATTCCTTGTCATCGT-3′ (reverse), for Δ97–180: 5′-CATGTGCAGCTTGCATACGA-3′ (forward), and 5′-TAAGTTGTGGGCCGTGGTTA-3′ (reverse), for Δ181-272: 5′-TTTTATCTCCCGACGACAGG-3′ (forward), and 5′-ACCACGTAAAGCCACAAGCT-3′ (reverse), for Δ273-341: 5′-TTAACGAGCAGAGGGAATGA-3′ (forward), and 5′-GTGATTATCCGAATTTCGGA-3′ (reverse), for Δ342-441: 5′-GACCTTGAAGAGGAAGAGGA-3′ (forward), and 5′-CTGAGAGCATGTCTGCTGGG-3′ (reverse), for Δ442-537: 5′-TAACTCGAGCATGCATCTAG-3′ (forward), and 5′-ACCAGACAGAGATGCTACTG-3′ (reverse). The C-terminal deletion mutants were generated using the following primers: for Δ442-470: 5′-GAGGCTCAGGACGCAAATCT-3′ (forward), and 5′- ACCAGACAGAGATGCTACTG-3′ (reverse), for Δ471-498: 5′-GAGGATGGGGAATTTTCAGA-3′ (forward), and 5′-AAGCCAGGCCTCAAATTCCT-3′ (reverse), for Δ499-537: 5′-GACCATGAAGGGGATGAGG-3′ (forward), and 5′-CGAACCATCCTCGTCCTCAT-3’ (reverse), for Δ512-537: 5′-TAACTCGAGCATGCATCTAG-3′ (forward), and 5′-GCTGTCAGACAGATCCA-3′ (reverse). The mutants harboring the serine-to-alanine substitution were prepared in the same fashion using the following primers: for S498A: 5′-GCGGAGGATGGGGAATTTTC-3′ (forward), and 5′- ACCATCCTCGTCCTCATCAC-3′ (reverse), for S504A: 5′- GCAGACCTGGATCTGTCTGA-3′ (forward), and 5′- AAATTCCCCATCCTCCGAAC-3′ (reverse), for S509A: 5′-GACAGCGACCATGAAGGGGA-3′ (forward), and 5′- AGCCAGATCCAGGTCTGAAA-3′ (reverse), for S511A: 5′- GCCGACCATGAAGGGGATGA-3′ (forward), and 5′- GTCAGACAGATCCAGGTCTG-3′ (reverse).

### Complementation Assay

To induce lytic replication in HEK293 cells carrying EBV BAC clones, cells were transfected with pcDNABZLF1 using the Neon transfection system (Invitrogen). At 72 h after transfection, cells and culture medium were collected, followed by freezing and thawing. After centrifugation at 13,000 rpm, the supernatants were mixed to Akata(-) cells for 3 h at room temperature with rotation, and the medium was replaced with fresh medium. At 48 h post-infection, cells were fixed with 1% formaldehyde, washed with PBS, and suspended in PBS. GFP-positive cells were counted using the FACSCalibur G5 system (Becton-Dickinson), according to the manufacturer’s instructions.

### Immunofluorescence Staining

HeLa cells were transfected with the indicated expression vectors using lipofectamine 2000 reagent (Invitrogen) and fixed with 70% ethanol. Cells were permeabilized using 0.1% Triton-X100, blocked using 10% normal goat serum (Funakoshi), and stained with anti-FLAG mouse antibody (Sigma), followed by washing and treatment with secondary antibody (Alexa 555 anti-mouse IgG, Invitrogen). AGS-EBV cells were transfected with BZLF1 and FLAG-BRRF2 using lipofectamine 2000 reagent. The cells were fixed with 70% ethanol on glass slides, permeabilized, and blocked. After staining with anti-FLAG mouse antibody and anti-BALF2 rabbit antibody, cells were reacted with secondary antibodies (Alexa Fluor 555 anti-mouse and anti-rabbit IgG, Invitrogen). B95-8 cells were transfected using the Neon transfection system (Invitrogen) and stained with mouse anti-BMRF1 antibody directly labeled with Alexa Fluor 488 using Zenon Alexa IgG labeling reagent (Invitrogen). After washing with PBS, cells were mounted using ProLong Diamond Antifade Mountant with DAPI (Invitrogen). Confocal microscopy (LSM880, Zeiss) was used to analyze the samples.

### Immunoblotting

For conventional SDS-PAGE, HEK293 cells carrying each BAC clone were transfected with the BZLF1 and BRRF2 mutant vectors using the Neon transfection system to induce lytic infection. After 2 days, cells were washed with PBS and lysed using sample buffer for SDS-PAGE. Electrophoresis, transfer, and staining were carried out as described previously (Narita et al., 2015).

### Separation and Detection of Phosphoprotein

HEK293 cells latently infected with BRRF2-knockout EBV were transfected with pcDNABZLF1 together with expression vector for wild-type or mutant FLAG-tagged BRRF2. After one day, cells were lysed in phosphatase buffer (Santa Cruz). Lysed samples were reacted with or without Lambda phosphatase (Santa Cruz). Protein samples were separated on a 7.5% Phos-tag gel (Wako chemicals). After electrophoresis, the Phos-tag gel was washed with transfer buffer containing EDTA to remove zinc ions from the gel, according to the manufacturer’s instructions, followed by immunoblotting as specified above.

## Results

### Functional Domains of BRRF2

To identify the functional domain(s) of BRRF2 involved in virus production, we determined whether BRRF2 mutants lacking various amino acid regions could complement the decreased viral titer caused by disruption of the BRRF2 gene. We prepared BRRF2 mutant vectors, each lacking ∼90 amino acids in various regions, as depicted in **Figure 1A**. These FLAG-tagged deletion mutant vectors were transfected into HEK293 cells latently infected with the BRRF2-knockout EBV BAC clone established previously (Watanabe et al., 2015b). Wild-type BRRF2 increased the virus yield of the BRRF2-knockout virus by two- or three-fold (**Figure 1B**) (Watanabe et al., 2015b). Likewise, mutants lacking the central regions of BRRF2 (Δ181–272, Δ273–341, and Δ342–441) successfully restored the impaired viral titer (**Figure 1B**). However, BRRF2 mutants lacking residues 1–180 at the N-terminus (Δ1–96 and Δ97–180) and 442–537 at the C-terminus (Δ442–537) failed to restore the reduced virus yield (**Figure 1B**). This result suggested that EBV BRRF2 has two important domains in both the N- and C-terminal regions (residues 1–180 and 442–537) required for efficient production of virus progeny.

### Cytoplasmic Localization of BRRF2 Requires the C-Terminal Region

We demonstrated previously that BRRF2 is localized predominantly in the cytoplasm of infected and transfected cells (Watanabe et al., 2015b). To investigate whether BRRF2 deletions alter the intracellular localization of the protein, we used immunofluorescence assays to observe the distribution of the mutant proteins. HeLa cells were transfected with vectors harboring the deletion mutants (shown in **Figure 1A**), stained with anti-FLAG antibody, and observed by confocal microscopy. Although BRRF2 lacking the N-terminal or central regions was localized in the cytoplasm in a manner similar to that of wild-type BRRF2, C-terminally truncated BRRF2 (Δ442–537) was found not only in the cytoplasm but also in the nucleus (**Figure 1C**). This result indicates that the C-terminal region of BRRF2 is required for its predominant localization in the cytoplasm, at least when produced in the absence of other viral proteins.

To analyze BRRF2 localization in the context of infection, we stained B95-8 and AGS-EBV cells after lytic induction (**Figure 1D**). The BMRF1 protein was also stained to monitor the lytic induction. Regardless of the cell type or presence of truncated BRRF2, BMRF1 protein was clearly localized in the nucleus as expected (**Figure 1D**). Whereas wild-type BRRF2 was detected mainly in the cytoplasm, the C-terminally truncated mutant (Δ442–537) was distributed in the nucleus and cytoplasm (**Figure 1D**). This result indicated that the BRRF2 C-terminal region is important for its predominant localization in the cytoplasm, even in the context of infection.

### Mutants Containing a Narrow Set of BRRF2 Deletions

Interestingly, residues 442–537 contain three stretches of acidic amino acid clusters, as shown in **Figure 1A** and **2A**. As several herpesvirus gene products, such as EBV BBLF1 (Chiu et al., 2012), retain acidic amino acid clusters crucial for localization in the cytoplasm (including the Golgi apparatus), we speculated that these acidic residues in BRRF2 might also play a role in cytoplasmic localization.

To narrow down the C-terminal residues of BRRF2 important for efficient progeny production and cytoplasmic distribution, we further constructed four additional deletion mutant vectors between residues 442 and 537 (Δ442–470, Δ471–498, Δ499–537, and Δ512–537), as shown in **Figure 2A**. Wild-type BRRF2 successfully augmented the viral titer, but the Δ442–537 mutant failed to do so (**Figure 2B**). However, all four deletion mutants of BRRF2 increased the yield almost as efficiently as did wild-type BRRF2 (**Figure 2B**). In addition, all four mutant proteins were localized in the cytoplasm, similar to the wild-type protein (**Figure 2C**). These results suggest the presence of redundant acidic amino acids in the BRRF2 protein.

### Phosphorylation of BRRF2 Serine 511 During the Lytic Cycle

Previous reports have shown that the tegument protein BRRF2 is phosphorylated in infected cells (Johannsen et al., 2004; Watanabe et al., 2015b). To identify the phosphorylation site of BRRF2, we used the phos-tag electrophoresis system, in which phosphate residues are bound by phos-tag molecules, thus retarding the mobility of phosphoproteins during electrophoresis (Kinoshita-Kikuta et al., 2007). Phosphoproteins treated with phosphatase migrate through the gel faster than those without phosphatase treatment.

**FIGURE 3:**
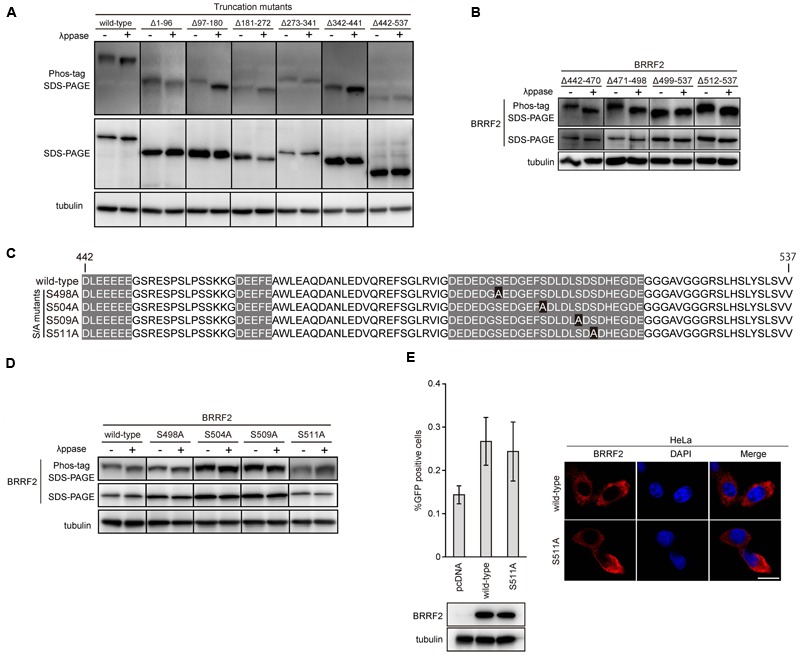
**Phosphorylation of BRRF2 at serine 511. (A)** Phosphorylation of BRRF2 at its C-terminus (residues 442–537). HEK293 cells carrying the BRRF2-knockout EBV BAC clone were transfected with BZLF1 and wild-type or BRRF2 deletion mutants (shown in **Figure 1A**) and harvested on day 2 with or without lambda phosphatase treatment. Protein samples were electrophoresed on a Phos-tag gel or by conventional SDS-PAGE for detection of BRRF2 and tubulin by immunoblotting. **(B)** Phosphorylation of BRRF2 within residues 499–511. Mutants containing a narrow set of BRRF2 deletions (shown in **Figure 2A**) were transfected, harvested, and blotted as in **(A)**. **(C)** Schematic diagram of EBV BRRF2 mutant vectors. Acidic cluster motifs are indicated with shadowing. Serine residues substituted with alanine residues were indicated with black background. **(D)** Phosphorylation of BRRF2 at serine 511. Mutants containing alanine substitution of serines 498, 504, 509, 511 (shown in **Figure 3C**) were transfected, harvested, and blotted as in **(A)**. **(E)** Phosphorylation of serine 511 did not influence the ability of BRRF2 to improve viral yield. HEK293 cells carrying the BRRF2-knockout EBV BAC clone were transfected with BZLF1 and the indicated BRRF2 vectors. Virus titer was determined as in **Figure 1B**. Protein levels were examined by immunoblotting. Intracellular localization of wild-type or S511A mutant of BRRF2 was examined by immunofluorescence in HeLa cells.

We first analyzed the phosphorylation state of BRRF2 mutants lacking various amino acid regions (see **Figure 1A**) with or without phosphatase treatment (**Figure 3A**). HEK293 cells expressing EBV BRRF2 lacking residues 442–537 migrated at the same speed in the phos-tag gel regardless of phosphatase treatment (**Figure 3A**, Δ442–537). In contrast, wild-type or other deletion mutant BRRF2 proteins (Δ1–96, Δ97–180, Δ181–272, Δ273–341, and Δ342–441) migrated faster when de-phosphorylated (**Figure 3A**). Thus, residues 442–537 contain phosphorylated amino acid.

Next, we examined the deletion mutants (Δ442–470, Δ471–498, Δ499–537, and Δ512–537) to determine which specific region is phosphorylated (see **Figure 2A**). BRRF2 Δ512–537 migrated faster when treated with phosphatase, whereas the mobility of BRRF2 Δ499–537 did not change in the presence or absence of phosphatase (**Figure 3B**), indicating that phosphorylated amino acid is between residues 499 and 511.

We thus created four mutants with alanine substitutions at each of the four serine residues in this region as shown in **Figure 3C** and evaluated whether these mutants were phosphorylated (**Figure 3D**). The results showed that BRRF2 S511A protein migrated similarly regardless of phosphatase treatment (**Figure 3D**), indicating that serine 511 undergoes phosphorylation.

We then subjected the BRRF2 S511A mutant to a complementation assay and found that this mutant restored the production of progeny as efficiently as did wild-type BRRF2 (**Figure 3E** left panels). In addition, BRRF2 S511A protein showed a similar localization pattern as that observed with wild-type BRRF2 (**Figure 3E** right panels). These results suggested that BRRF2 serine 511 is phosphorylated during lytic infection; however, this phosphorylation is not essential for efficient production of viral progeny.

### Detailed Analysis of Intercellular Localization of BRRF2

**FIGURE 4:**
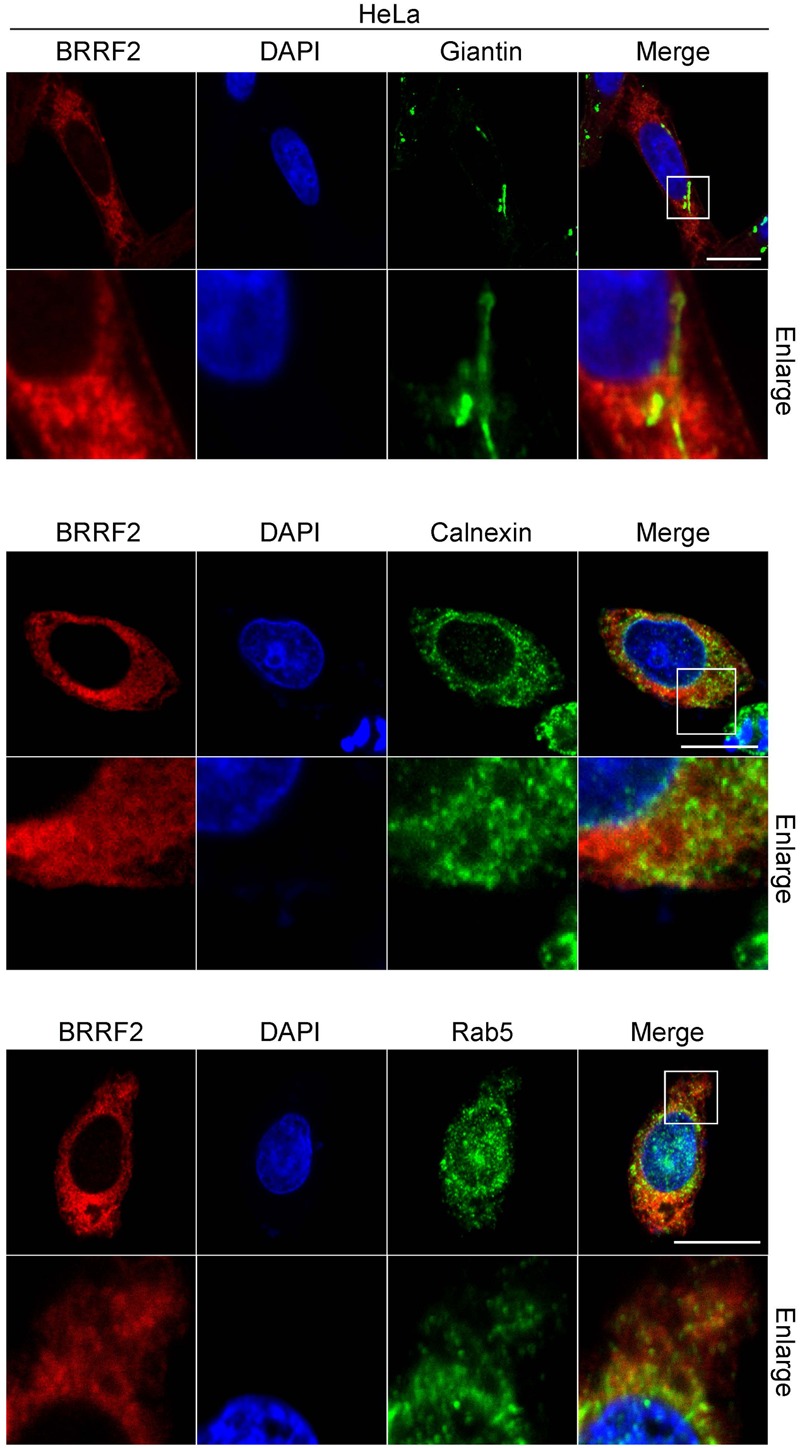
**Co-staining of BRRF2 with organelle markers.** HeLa cells were transfected with the vector for FLAG-tagged wild-type BRRF2. Cells were stained with ant-FLAG (BRRF2, red), Giantin (upper panels, green), Calnexin (middle panels, green), and Rab5 (lower panels, green) antibodies and DAPI (blue) and analyzed by using confocal laser microscopy. The white bar indicates 10 μm.

To clarify the localization of BRRF2 in the cytoplasm, we transfected HeLa cells with wild-type BRRF2 and co-stained with markers of cytoplasmic organelles (**Figure 4**). The pattern of Giantin, which is known to localize in the Golgi apparatus, was different from that of BRRF2 although a part of BRRF2 protein might colocalize (**Figure 4** upper panels). The Calnexin resides in endoplasmic reticulum (ER). It might colocalize with BRRF2 protein, albeit only partly (**Figure 4** middle panels). Interestingly, localization of BRRF2 matched at least partially with Rab5, which is a marker of endosomes, especially of early endosome (**Figure 4** lower panels). Therefore, unlike BBLF1 (Chiu et al., 2012), BRRF2 likely localizes more efficiently to endosomes, than Golgi apparatus and ER.

## Discussion

We investigated the domains of the BRRF2 protein required for efficient production of progeny by analyzing deletion mutants of BRRF2. We found that the amino acids in the N-terminal (1–180) and C-terminal (442–537) regions of BRRF2 are crucial for efficient EBV multiplication (**Figure 1**). Interestingly, the C-terminal portion of BRRF2, which contains three stretches of acidic residues, appears to be crucial for appropriate cytoplasmic localization of the protein and optimal production of virus particles (**Figure 1**). A similar acidic cluster motif was reported to play an essential role in the cytoplasmic trafficking of Furin, a cellular protease that cleaves many proteins in the secretory pathway (Thomas, 2002). Furin has two serine residues (773 and 775) within its acidic motif that are phosphorylated by casein kinase 2 (CK2). When phosphorylated, the acidic cluster of Furin consolidates binding to phospho-furin acidic cluster sorting protein-1 (PACS-1), which then directs Furin to the TGN (Wan et al., 1998). After this finding, many cellular and viral proteins were found to have acidic clusters (and in some cases, phosphorylation motifs within the acidic cluster) that are crucial for cytoplasmic localization. For example, HIV Nef binds to PACS-1 through its acidic cluster, resulting in relocalization of cell surface MHC class I molecules to the TGN (Piguet et al., 2000). Many herpesvirus tegument proteins and glycoproteins contain acidic amino acid clusters. HSV VP22 retains an acidic cluster that is essential for TGN localization and incorporation into virions (O’Regan et al., 2010). HSV UL11 and its homologs, HCMV UL99 and EBV BBLF1, are localized to the TGN via their acidic clusters (Loomis et al., 2001; Jones and Lee, 2004; Chiu et al., 2012). The acidic cluster motifs of HCMV gB interact with PACS-1, and this interaction facilitates its localization to the TGN (Crump et al., 2003). KSHV tegument protein ORF45 also harbors an acidic patch, through which ORF45 interacts with another tegument protein ORF36 (Avey et al., 2016). Similar to Furin, EBV BRRF2 has a phosphorylation motif in one of the acidic clusters (**Figure 3**). However, in our study, substitution of serine 511 with an alanine did not alter the intracellular localization, indicating that the phosphorylation-dependent mechanism may not be applicable to BRRF2. KSHV ORF48, a homolog of EBV BRRF2, contains conserved acidic cluster motifs, which may contribute to its cytoplasmic localization in a manner similar to that of EBV BRRF2.

In addition to the acidic clusters and CK2-phosphorylated residues in the clusters, a dileucine-based motif (LL or replacement of the second leucine with isoleucine, valine, or methionine) and a tyrosine-based motif (YXXF, where X is any amino acid and F is an amino acid with a bulky hydrophobic side chain) have been reported to control localization in the TGN of at least some proteins (Gu et al., 2001). Typically, the LL-based motif is surrounded by or adjacent to acidic clusters. EBV BRRF2 contains four LL, one LI, three LV, and one LM motifs, but none of the possible LL motifs are within or around the acidic clusters; all possible LL motifs are actually in the N-terminal half of the protein. We identified a potential tyrosine-based motif (Y_150_EIP) in the N-terminal portion of BRRF2. However, deletion of the N-terminal region of BRRF2 had little or no effect on its localization (**Figure 1C**), indicating that these residues probably do not function as TGN-targeting motifs.

Although the mislocalization to the nucleus and little or no improvement of viral yield by BRRF2 were observed in the case of Δ442–537 mutant (**Figure 1**), the change in localization of the protein may not necessarily be the direct cause of lower titer. To further clarify the consequences, we need to test BRRF2 mutants with smaller deletions. However, such effort was not successful because the mutants with smaller deletions all acted like wild-type BRRF2 (**Figure 2**).

Unlike other cellular or viral proteins bearing acidic cluster motifs, we lastly showed that BRRF2 merged, to some extent, with Rab5, an endosome marker. It is speculated that presence of acidic clusters (the possible targeting motif for TGN) in the BRRF2 protein still makes sense because TGN and endosomes have intricate communications. Endosomal distribution of a tegument protein BRRF2 suggests that the secondary envelopment of EBV may take place at the endosome, where viral nucleocapsids incorporate tegument proteins and wear envelope.

In addition to the C-terminal region, N-terminal 180 residues of BRRF2 were also involved in the restoration of viral yield, without obviously affecting its localization (**Figure 1**). We speculate that the N-terminal portion of BRRF2 protein may interact with other viral tegument or capsid protein while the C-terminal domain may aid cytoplasmic membrane targeting. Such bipolar interaction might somehow promote efficient tegumentation and/or secondary envelopment on the surface of cytoplasmic membrane, such as TGN or endosome. Interestingly, we hear that ORF48 of murine gammaherpesvirus-68 (homolog of EBV BRRF2) may interact with a capsid protein (Dr. Ohno (University of the Ryukyus), personal communication).

The molecular mechanisms by which BRRF2 potentiates the production of EBV progeny remain unclear; however, our data in this and previous reports (Watanabe et al., 2015b) suggest that BRRF2 enhances secondary envelopment and efficient tegumentation in the cytoplasm. Further studies on BRRF2 and other tegument and envelope proteins are required to clarify EBV tegumentation and secondary envelopment processes in greater detail.

## Author Contributions

TW and TM designated the experiments; TW, KS, MY, HM, carried out experiments; YS, FG, HK, TM supervised and discussed the experiments and data; TW and TM prepared the manuscript.

## Conflict of Interest Statement

The authors declare that the research was conducted in the absence of any commercial or financial relationships that could be construed as a potential conflict of interest.

## References

[B1] Avey D. Tepper S. Pifer B. Bahga A. Williams H. Gillen J. (2016). Discovery of a coregulatory interaction between Kaposi’s sarcoma-associated herpesvirus ORF45 and the viral protein kinase ORF36. *J. Virol.* 90 5953–5964. 10.1128/JVI.00516-16 27099309 PMC4907238

[B2] Chiu Y. F. Sugden B. Chang P. J. Chen L. W. Lin Y. J. Lan Y. C. (2012). Characterization and intracellular trafficking of Epstein-Barr virus BBLF1, a protein involved in virion maturation. *J. Virol.* 86 9647–9655. 10.1128/JVI.01126-12 22740416 PMC3446546

[B3] Crump C. M. Hung C. H. Thomas L. Wan L. Thomas G. (2003). Role of PACS-1 in trafficking of human cytomegalovirus glycoprotein B and virus production. *J. Virol.* 77 11105–11113. 10.1128/JVI.77.20.11105-11113.2003 14512558 PMC224974

[B4] Delecluse H. J. Hilsendegen T. Pich D. Zeidler R. Hammerschmidt W. (1998). Propagation and recovery of intact, infectious Epstein-Barr virus from prokaryotic to human cells. *Proc. Natl. Acad. Sci. U.S.A.* 95 8245–8250. 10.1073/pnas.95.14.8245 9653172 PMC20961

[B5] Djavadian R. Chiu Y. F. Johannsen E. (2016). An Epstein-Barr virus-encoded protein complex requires an origin of lytic replication in cis to mediate late gene transcription. *PLoS Pathog.* 12:e1005718 10.1371/journal.ppat.1005718 PMC4922670 27348612

[B6] Gruffat H. Marchione R. Manet E. (2016). Herpesvirus late gene expression: a viral-specific pre-initiation complex is key. *Front. Microbiol.* 7:869 10.3389/fmicb.2016.00869 PMC4893493 27375590

[B7] Gu F. Crump C. M. Thomas G. (2001). Trans-Golgi network sorting. *Cell. Mol. Life. Sci.* 58 1067–1084. 10.1007/PL00000922 11529500 PMC1424219

[B8] Hammerschmidt W. Sugden B. (2013). Replication of Epstein-Barr viral DNA. *Cold Spring Harb. Perspect. Biol.* 5:a013029 10.1101/cshperspect.a013029 PMC3579399 23284049

[B9] Johannsen E. Luftig M. Chase M. R. Weicksel S. Cahir-McFarland E. Illanes D. (2004). Proteins of purified Epstein-Barr virus. *Proc. Natl. Acad. Sci. U.S.A.* 101 16286–16291. 10.1073/pnas.0407320101 15534216 PMC528973

[B10] Johnson D. C. Baines J. D. (2011). Herpesviruses remodel host membranes for virus egress. *Nat. Rev. Microbiol.* 9 382–394. 10.1038/nrmicro2559 21494278

[B11] Jones T. R. Lee S. W. (2004). An acidic cluster of human cytomegalovirus UL99 tegument protein is required for trafficking and function. *J. Virol.* 78 1488–1502. 10.1128/JVI.78.3.1488-1502.2004 14722304 PMC321399

[B12] Katsumura K. R. Maruo S. Wu Y. Kanda T. Takada K. (2009). Quantitative evaluation of the role of Epstein-Barr virus immediate-early protein BZLF1 in B-cell transformation. *J. Gen. Virol.* 90 2331–2341. 10.1099/vir.0.012831-0 19553389

[B13] Kawashima D. Kanda T. Murata T. Saito S. Sugimoto A. Narita Y. (2013). Nuclear transport of Epstein-Barr virus DNA polymerase is dependent on the BMRF1 polymerase processivity factor and molecular chaperone Hsp90. *J. Virol.* 87 6482–6491. 10.1128/JVI.03428-12 23552409 PMC3648106

[B14] Kinoshita-Kikuta E. Aoki Y. Kinoshita E. Koike T. (2007). Label-free kinase profiling using phosphate affinity polyacrylamide gel electrophoresis. *Mol. Cell. Proteomics* 6 356–366. 10.1074/mcp.T600044-MCP200 17088264

[B15] Lieberman P. M. (2013). Keeping it quiet: chromatin control of gammaherpesvirus latency. *Nat. Rev. Microbiol.* 11 863–875. 10.1038/nrmicro3135 24192651 PMC4544771

[B16] Loomis J. S. Bowzard J. B. Courtney R. J. Wills J. W. (2001). Intracellular trafficking of the UL11 tegument protein of herpes simplex virus type 1. *J. Virol.* 75 12209–12219. 10.1128/JVI.75.24.12209-12219.2001 11711612 PMC116118

[B17] Mettenleiter T. C. Klupp B. G. Granzow H. (2006). Herpesvirus assembly: a tale of two membranes. *Curr. Opin. Microbiol.* 9 423–429. 10.1016/j.mib.2006.06.013 16814597

[B18] Murata T. (2014). Regulation of Epstein-Barr virus reactivation from latency. *Microbiol. Immunol.* 58 307–317. 10.1111/1348-0421 24786491

[B19] Murata T. Isomura H. Yamashita Y. Toyama S. Sato Y. Nakayama S. (2009). Efficient production of infectious viruses requires enzymatic activity of Epstein-Barr virus protein kinase. *Virology* 389 75–81. 10.1016/j.virol.2009.04.007 19427010

[B20] Murata T. Tsurumi T. (2014). Switching of EBV cycles between latent and lytic states. *Rev. Med. Virol.* 24 142–153. 10.1002/rmv.1780 24339346

[B21] Narita Y. Sugimoto A. Kawashima D. Watanabe T. Kanda T. Kimura H. (2015). A herpesvirus specific motif of Epstein-Barr virus DNA polymerase is required for the efficient lytic genome synthesis. *Sci. Rep.* 5:11767 10.1038/srep11767 PMC4485236 26123572

[B22] O’Regan K. J. Brignati M. J. Murphy M. A. Bucks M. A. Courtney R. J. (2010). Virion incorporation of the herpes simplex virus type 1 tegument protein VP22 is facilitated by trans-Golgi network localization and is independent of interaction with glycoprotein E. *Virology* 405 176–192. 10.1016/j.virol.2010.06.007 20580397 PMC3466811

[B23] Piguet V. Wan L. Borel C. Mangasarian A. Demaurex N. Thomas G. (2000). HIV-1 Nef protein binds to the cellular protein PACS-1 to downregulate class I major histocompatibility complexes. *Nat. Cell Biol.* 2 163–167. 10.1038/35023643 10707087 PMC1475706

[B24] Qi J. Han C. Gong D. Liu P. Zhou S. Deng H. (2015). Murine gammaherpesvirus 68 ORF48 is an RTA-responsive gene product and functions in both viral lytic eplication and latency during in vivo infection. *J. Virol.* 89 5788–5800. 10.1128/JVI.00406-15 25762743 PMC4442421

[B25] Sugimoto A. Sato Y. Kanda T. Murata T. Narita Y. Kawashima D. (2013). Different distributions of Epstein-Barr virus early and late gene transcripts within viral replication compartments. *J. Virol.* 87 6693–6699. 10.1128/JVI.00219-13 23552415 PMC3676136

[B26] Thomas G. (2002). Furin at the cutting edge: from protein traffic to embryogenesis and disease. *Nat. Rev. Mol. Cell Biol.* 3 753–766. 10.1038/nrm934 12360192 PMC1964754

[B27] Tsurumi T. Fujita M. Kudoh A. (2005). Latent and lytic Epstein-Barr virus replication strategies. *Rev. Med. Virol.* 15 3–15. 10.1002/rmv.441 15386591

[B28] Wan L. Molloy S. S. Thomas L. Liu G. Xiang Y. Rybak S. L. (1998). PACS-1 defines a novel gene family of cytosolic sorting proteins required for trans-Golgi network localization. *Cell* 94 205–216. 10.1016/S0092-8674(00)81420-8 9695949

[B29] Watanabe T. Narita Y. Yoshida M. Sato Y. Goshima F. Kimura H. (2015a). The Epstein-Barr virus BDLF4 gene is required for efficient expression of viral late lytic genes. *J. Virol.* 89 10120–10124. 10.1128/JVI.01604-15 26202235 PMC4577904

[B30] Watanabe T. Tsuruoka M. Narita Y. Katsuya R. Goshima F. Kimura H. (2015b). The Epstein-Barr virus BRRF2 gene product is involved in viral progeny production. *Virology* 484 33–40. 10.1016/j.virol.2015.05.010 26057150

[B31] Young L. S. Yap L. F. Murray P. G. (2016). Epstein-Barr virus: more than 50 years old and still providing surprises. *Nat. Rev. Cancer* 16 789–802. 10.1038/nrc.2016.92 27687982

